# Fast and expensive (PCR) or cheap and slow (culture)? A mathematical modelling study to explore screening for carbapenem resistance in UK hospitals

**DOI:** 10.1186/s12916-018-1117-4

**Published:** 2018-08-16

**Authors:** Gwenan M. Knight, Eleonora Dyakova, Siddharth Mookerjee, Frances Davies, Eimear T. Brannigan, Jonathan A. Otter, Alison H. Holmes

**Affiliations:** 1 National Institute of Health Research Health Protection Research Unit in Healthcare Associated Infections and Antimicrobial Resistance, Imperial College London, Commonwealth Building, Hammersmith Campus, Imperial College London, Du Cane Road, London, W12 0NN UK; 2 Infectious Diseases and Immunity, Commonwealth Building, Hammersmith Campus, Imperial College London, Du Cane Road, London, W12 0NN UK; 3 Imperial College Healthcare NHS Trust, London, UK

**Keywords:** Carbapenem resistance, Screening algorithms, Mathematical modelling, PCR, Culture

## Abstract

**Background:**

Enterobacteriaceae are a common cause of hospital infections. Carbapenems are a clinically effective treatment of such infections. However, resistance is on the rise. In particular, carbapenemase-producing carbapenem-resistant Enterobacteriaceae (CP-CRE) are increasingly common. In order to limit spread in clinical settings, screening and isolation is being recommended, but many different screening methods are available. We aimed to compare the impact and costs of three algorithms for detecting CP-CRE carriage.

**Methods:**

We developed an individual-based simulation model to compare three screening algorithms using data from a UK National Health Service (NHS) trust. The first algorithm, “Direct PCR”, was highly sensitive/specific and quick (half a day), but expensive. The second, “Culture + PCR”, was relatively sensitive/specific but slower, requiring 2.5 days. A third algorithm, “PHE”, repeated the “Culture + PCR” three times with an additional PCR. Scenario analysis was used to compare several levels of CP-CRE prevalence and coverage of screening, different specialities as well as isolation strategies. Our outcomes were (1) days that a patient with CP-CRE was not detected and hence not isolated (“days at risk”), (2) isolation bed days, (3) total costs and (4) mean cost per CP-CRE risk day averted per year. We also explored limited isolation bed day capacity.

**Results:**

We found that although a Direct PCR algorithm would reduce the number of CP-CRE days at risk, the mean cost per CP-CRE risk day averted per year was substantially higher than for a Culture + PCR algorithm. For example, in our model of an intensive care unit, during a year with a 1.6% CP-CRE prevalence and 63% screening coverage, there were 508 (standard deviation 15), 642 (14) and 655 (14) days at risk under screening algorithms Direct PCR, Culture + PCR and PHE respectively, with mean costs per risk day averted of £192, £61 and £79. These results were robust to sensitivity analyses.

**Conclusions:**

Our results indicate that a Culture + PCR algorithm provides the optimal balance of cost and risk days averted, at varying isolation, prevalence and screening coverage scenarios. Findings from this study will help clinical organisations determine the optimal screening approach for CP-CRE, balancing risk and resources.

**Electronic supplementary material:**

The online version of this article (10.1186/s12916-018-1117-4) contains supplementary material, which is available to authorized users.

## Background

Carbapenem antibiotics are clinically effective and well tolerated for the treatment of antibiotic-resistant Gram-negative bacteria and hence extremely important for tackling life-threatening infections in UK hospitals [1]. The most common cause of blood stream infections in England is Gram-negative bacteria, specifically Enterobacteriaceae [2]. The number of infections with carbapenem-resistant Enterobacteriaceae (CRE) is on the rise [3]. These infections lead to increased morbidity, mortality and cost [4].

Carbapenemase-producing carbapenem-resistant Enterobacteriaceae (CP-CRE) are effectively a sub-population of CRE which represent a further threat, because the genes encoding the mechanisms of resistance (a carbapenemase) can be transferred between bacterial species and confer elevated levels of resistance compared with other mechanisms of carbapenem resistance [5, 6]. CP-CRE have shown a notable rise in the number of cases over the last decade in England [7]. They were close to absent in 2006 but have since increased to more than 2500 isolates being referred to the national reference laboratory in 2016 [8]. Outbreaks have also been detected in some centres [9], and others report endemic CP-CRE [10]. As a result, increasing numbers of patients have extremely limited therapy options; thus preventive infection control practices such as active screening and single room isolation play an even more important part in our clinical settings [11]. CP-CRE colonisation has been shown to correlate positively with the incidence of infection attributed to CP-CRE organisms, particularly in an intensive care unit (ICU) setting [12]. There are currently no accepted decolonisation protocols for CP-CRE organisms; hence early detection of asymptomatic carriage and isolation are key tools to prevent transmission. However, there is insufficient data on the effectiveness or cost-effectiveness of various CP-CRE screening algorithms.

US (Centers for Disease Control and Prevention, CDC), European (European Centre for Disease Prevention and Control, ECDC) and UK (Public Health England, PHE) organisations have published guidance highlighting the importance of patient screening for CP-CRE in order to identify the carriers and prevent subsequent infection and spread [13, 14]. The European Society of Clinical Microbiology and Infectious Diseases (ESCMID) recommended patient screening on admission in both endemic and epidemic settings as well as pre-emptive isolation in a single room in an epidemic setting [15]. However, there are a number of challenges associated with this, such as the high cost of patient screening on admission, which may not always be optimal due to the wide range of prevalence on admission in different areas [16, 17]. Moreover, some CP-CRE screening methods take up to 48 h to give a result. Hence, pre-emptive isolation may not be an option, as it could result in a high number of patients being isolated unnecessarily for a prolonged period of time [18], nor may it be feasible given the limited availability of isolation facilities.

The most common existing CP-CRE screening methods include conventional culture-based approaches, which have good sensitivity and specificity but take several days to return a result [13], and molecular polymerase chain reaction (PCR)-based methods, which are much faster, at least as sensitive, but substantially more expensive [19]. Importantly, CRE is defined phenotypically, whereas CP-CRE is a genotypic phenomenon most commonly determined by means of a molecular-based test. Whilst PCR-based methods can only detect known carbapenemase-encoding genes that they are designed to detect [20], culture-based methods do not detect non-expressed genetic mechanisms [21]. This suggests that a combination of culture- and PCR-based tests should be used in order to detect all phenotypic resistance and also to confirm the underlying genetic mechanisms.

Mathematical models, often used in the field of infectious diseases, provide the ideal platform from which to simulate a range of laboratory screening options to detect CP-CRE. Their use in the field of healthcare-associated infections is well documented, with previous models of antibiotic-resistant Gram-negatives mostly focused on the ICU [22–24] to evaluate interventions and screening algorithms [25].

Our aim was to compare the impact of different currently used screening algorithms for CP-CRE using data from Imperial College Healthcare NHS Trust (ICHNT) using a newly designed mathematical model. By comparing different scenarios, parameterised by data from a group of London teaching hospitals, we were able to explore the predicted clinical impact and the comparative cost of different molecular- and culture-based screening tools. This will help to inform both ICHNT and other hospital trusts as to which screening methods to use in clinical settings to help combat this increasing threat.

## Methods

### Data

ICHNT implemented a combination of universal screening for all admissions to certain high-risk specialities and risk factor-based screening for all other admissions in June 2015. Universal screening was implemented in the ICU, renal, vascular and haematology in-patient wards. This group of patients accounts for nearly half of all trust admissions. We used this universal admission screening data over a 9-month period (June 2015–March 2016) to calculate the prevalence of CP-CRE carriage at admission and screening coverage levels (Table 1). For the purpose of the prevalence calculation, each patient was only included once, despite the fact that most patients were screened multiple times during their hospital stay. Length of stay (LoS) distributions by CRE status (no carriage vs. carriage) were gathered for each speciality separately (Table 1 and Additional file 1: Table S1). We assumed that those patients with colonisation due to CRE-producing carbapenemases (CP-CRE) or non-carbapenemase-producing CRE (NCP-CRE) could be grouped as having the same LoS distribution.

**Table 1:** Parameter table. All parameters were estimated using ICHNT data

Parameter	Description		Value	References and notes
CP-CRE prevalence at admission	ICU		1.6% (16/1007)	Calculated from universal screening data of a total of 2870 patients, over a 9-month period
	Renal		1.9% (16/858)	
	Vascular		0.4% (2/541)	
	Haematology		1.3% (6/464)	
Coverage of initial admission screening	ICU		63.0%	
	Renal		67.0%	
	Vascular		48.0%	
	Haematology		68.0%	
Number of speciality beds	ICU		112	Sum of all wards in each speciality as in March 2016
	Renal		71	
	Vascular		65	
	Haematology		66	
Length of stay (mean/median)	ICU	S	7.9/4.0	Taken from speciality data and based on initial screening result
		CRE	15.9/10.0	
	Renal	S	7.8/5.0	
		CRE	15.5/12.0	
	Vascular	S	6.2/4.0	
		CRE	12.4/7.0	
	Haematology	S	9.6/5.0	
		CRE	19.6/9.0	
Time to result (days)	Culture		2	For single component test
	PHE PCR		7^a^	
	PCR		0.5	
	(A) Direct PCR		0.5	For complete algorithm
	(B) Culture + PCR		2.5	
	(C) PHE		13	

S patient was carrying no Enterobacteriaceae or Enterobacteriaceae susceptible to carbapenems, CRE patient was carrying Enterobacteriaceae resistant to carbapenems. These data come from the patients identified as carriers using the current ICHNT screening procedure

^a^Accounts for the PHE workload and specimen transportation

We used data from ICHNT to parameterise the costs of our screening tools (Additional file 1: Table S2). We performed an economic evaluation from the hospital perspective, following the Consolidated Health Economic Evaluation Reporting Standards (CHEERS) guidelines [26]. Over the time period investigated here, no charge was applied for additional diagnostic work performed at PHE. The cost of one isolation bed day was composed of a daily cost of £20.33 (£20 for gloves and aprons, £0.33 for infectious waste stream) and a one-off cost from stock disposal of £113 (£385 for the ICU) incurred at the time of patient discharge [9]. The time horizon was 1 year with no discount rate.

### Diagnostic algorithms

**Fig. 1:**
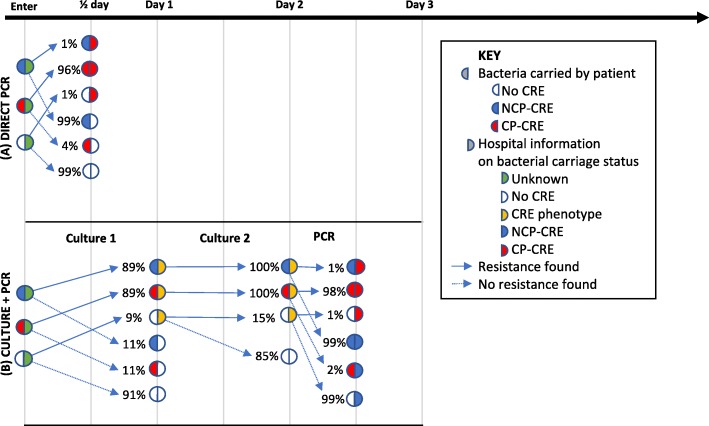
Details of the timings of the first two screening algorithms. The third screening algorithm (PHE) is shown in detail in Additional file 1: Figure S1. It is a combination of three (B) Culture + PCR tests with a further highly accurate PHE PCR test

Three diagnostic algorithms to detect CP-CRE were compared. The first (A) is a PCR test direct from a sample swab, the second (B) represents culture followed by carbapenemase confirmation by PCR (the current ICHNT hospital-based protocol), and the third (C) repeats (B) three times, with an additional PCR performed at the PHE national reference laboratory to confirm a lack of CRE. All sensitivity and specificity values are provided in Table 2. The details of the first two algorithms are shown in Fig. 1 (all algorithms are detailed in Additional file 1: Figure S1).

**Table 2:** Details of tests used in the algorithms

Test	Negative result	Sensitivity	Specificity	References and notes	Screening algorithm		
					(A) Direct PCR	(B) Culture + PCR	(C) PHE
PCR from swab	S	96%	99%	Tato et al., 2016 [27]	x		
ICHNT PCR	NCP-CRE^a^	98%	99%	ICHNT data		x	x
Culture (1)	S	89%	91%	ICHNT data		x	x
Culture (2)	NCP-CRE	100%	85%	ICHNT data		x	x
PHE PCR	NCP-CRE^a^	100%	100%	Assumed optimal			x

Sensitivity is the probability that the test detects resistance given that the patient carries resistant Enterobacteriaceae. Specificity is the probability of a negative test for resistance given that the patient does not carry resistant bacteria

^a^As both of these PCR tests are on samples that have shown to be culture positive for CRE, they are classified as NCP-CRE if the PCR test for CPE is negative

In detail, (A) “Direct PCR”, is a single, in-house PCR direct from a screening sample. It is a quick test, which would dramatically reduce detection time by giving results within half a day. We used estimates from the literature [27] to give a sensitivity and specificity of this test (Table 2).

The second algorithm (B) represents the current ICHNT protocol of “Culture + PCR”. The culture part has two stages: a screening swab plated onto chromogenic media (Colorex™ mSuperCARBA™, E&O Laboratories, Bonnybridge, UK), followed by suspicious colonies being tested for antimicrobial susceptibility (EUCAST disc diffusion). Those colonies with defined antimicrobial resistance profiles are tested using the ICHNT in-house PCR for carbapenemase gene detection (Xpert® Carba-R, Cepheid Inc., Sunnyvale, CA, USA). The genes targeted in this kit are *KPC*, *NDM*, *VIM*, *OXA-48* and *IMP* [28]. Sensitivity and specificity values were estimated from ICHNT data (Table 2). Each culture stage takes a day, whilst the PCR takes half a day (Table 1, Fig. 1).

The third algorithm (C) “PHE”, is aligned with the PHE recommendation of three sequential screens, each separated by 48 h to test for CRE carriage. Three culture tests, paired with ICHNT PCR, are performed across the whole population. In addition, a confirmatory PHE PCR test is performed for any suspicious colonies from culture. The PHE PCR was assumed to have a higher sensitivity than the ICHNT PCR. As above, sensitivity and specificity values were estimated from ICHNT data where possible (Table 2), and algorithm C takes 6.5 days to perform the three within-hospital screening tests, with a further 7 days for a PCR result from PHE (Table 1). This latter PCR test is unlikely to have an impact on our results, but it is included to reflect the current recommended algorithm and, due to the increased sensitivity, it may detect further colonisation.

### Scenarios

We considered four main scenarios including a baseline scenario parameterised to represent the ICHNT ICU with 100% screening coverage. This baseline scenario was then modified to include (1) an ICHNT ICU with screening coverage at < 100% (63%, the screening coverage in 2015–2016), (2) 63% screening coverage and high CP-CRE prevalence (20%) and (3) high (20%) CP-CRE prevalence. The high CP-CRE prevalence (20%) was used to represent a potentially catastrophic though not unrealistic scenario, as 2014 European average CRE prevalence levels were already 7.3% [29]. The corresponding prevalence of NCP-CRE was set at 5%, making overall CRE prevalence 25% in the high prevalence scenarios. In Additional file 1, we also provide results for the three other high-risk specialities (renal, vascular and haematology).

### Isolation strategies

We considered one main isolation strategy: (1) only those with confirmed CP-CRE were isolated (matching current ICHNT practice). In Additional file 1, we also provide results for (2) isolating no one and (3) isolating all patients with confirmed CRE (NCP- and CP-CRE). We did not consider any pre-emptive isolation strategies. In our main analysis, we report the number of isolation days required, making no assumptions about adherence to isolation policy or availability of isolation beds.

### Mathematical model

**Fig. 2:**
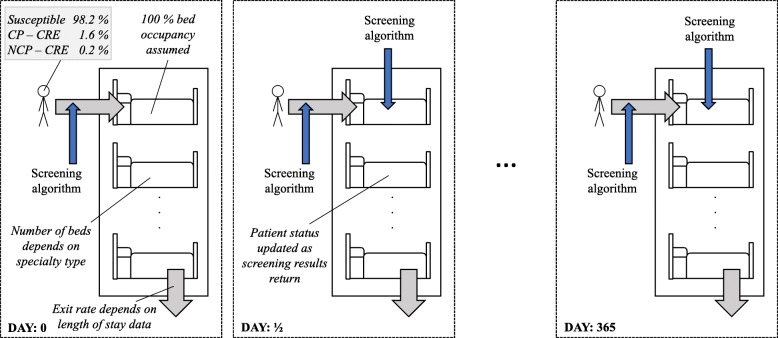
Underlying model structure. Incoming prevalence varies by scenario and speciality. Here, Susceptible refers to the patient carrying no Enterobacteriaceae or Enterobacteriaceae that are susceptible to carbapenems. As the ward is assumed to always be full, the rate at which patients enter is equal to the exit rate, which is the inverse of the length of stay. Screening compliance is used to determine how many of the patients are screened when they enter the speciality and during their stay (depending on the algorithm)

We constructed a stochastic (random) individual-based model of patients with or without CP-CRE carriage in hospital specialities (Fig. 2). This model captured four key parameters: (1) the rates at which patients exit the hospital (based on ICHNT LoS data and independent of screening status), (2) incoming CP-CRE prevalence rates, (3) time taken for a finalised result and (4) the efficacy of the screening tests (see screening algorithms in Table 2). The model was built to consider each speciality separately, allowing for patients to stay for an appropriate LoS and then replacing those who exit with new patients, screened on entry at a certain compliance level, making the speciality full at all times (Fig. 2).

The model was parameterised using data from ICHNT (Table 1). A stochastic model formulation was used to account for the small population sizes. The stochastic parameters included within the model were (1) the chance of being colonised with CRE (CP-CRE, NCP-CRE or no CRE) on entry, (2) the chance of being tested (based on screening coverage levels), (3) the chance of being detected (using the efficacy values as a probability) and (4) the LoS (selected with replacement from the data distribution).

We did not include secondary transmission in this model, as there is insufficient evidence on the levels of transmission of Enterobacteriaceae in a clinical setting and on the impact of isolation interventions on this transmission. Instead, the number of “days at risk” is a proxy for transmission level.

### Simulation

All simulations were performed in *R* [30]. All results are calculated from 100 runs, where one run captures the scenario for 1 year, with a time step of half a day. Error bars represent the standard error taken over the 100 runs. The single cost estimates were used to multiply the mean, minimum and maximum values from the 100 model outputs to give a range.

### Outcomes

Our primary outcome was the number of days at risk: the number of days that a patient with CP-CRE is not detected and hence not isolated.

Our secondary outcome was the total number of isolation days and the number of days those without CP-CRE were isolated (inappropriate isolation). These values were presented as totals, which we compared to the number of side rooms suitable for isolation at ICHNT in 2015, which totalled 60 [18, 25]. This results in a total number of isolation bed days available a year of 3650, 6205, 9125 and 2920 for ICU, renal, haematology and vascular specialities respectively.

Our third outcome was the total cost evaluation of each algorithm. The fourth outcome was the mean cost per risk day averted for patients with CP-CRE. This was obtained by dividing the mean total direct costs of each scenario (screening and isolation bed day costs) by the mean number of averted risk days (i.e. number of days that a patient with CP-CRE was isolated). At ICHNT, as screening algorithm (B) is the current protocol, we also calculated the average incremental cost associated with 1 additional averted risk day for screening algorithms (A) and (C) to inform direct decision making in our setting.

### Isolation bed day capacity effects

As previously outlined, there are currently a limited number of isolation beds at ICHNT. To account for this potential implementation barrier, we also calculated a “days at risk (adjusted for isolation capacity)” value. In this analysis, the isolation days that exceeded isolation capacity were added to the number of days at risk, so “days at risk (adjusted for isolation capacity)” included CP-CRE non-isolation because of (1) non-detection and (2) detection but a lack of available isolation bed. For this we assumed that isolation beds would only be used for CP-CRE.

We also recalculated the costs per risk day averted for patients with CP-CRE assuming that a risk day could only be averted if there was an isolation bed available.

### Sensitivity analysis

The four main scenarios (baseline ICU speciality with four different screening coverage and CRE prevalence levels) comprised our main sensitivity analysis. We also explored the impact of dramatically reducing the sensitivity of PCR to 60% to account for a change in the circulating genetic marker encoding the carbapenemase, such as the appearance of a novel or unusual carbapenemase, within the ICU ICHNT scenario (with 63% screening coverage).

## Results

### Outcomes

**Fig. 3:**
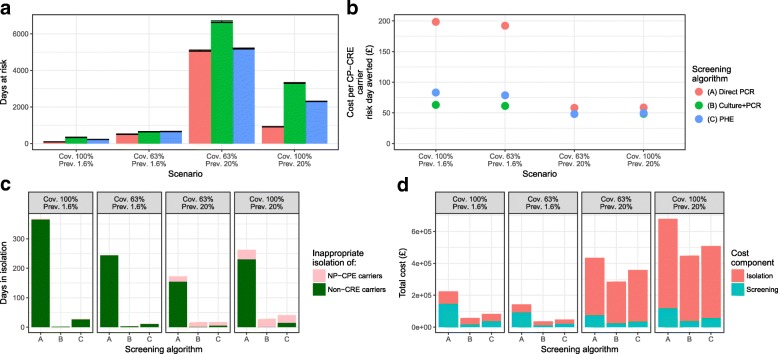
Results figure for the four ICU scenarios showing our two main outcomes: **a** number of days at risk and **b** cost per CP-CRE carrier risk day averted. Additional outcomes of inappropriate isolation days **c** and total costs (£) **d** are also shown. Error bars in **a** are standard error. No error bars are shown in **b**, as this is the ratio of means. Details of the errors for values in **c** and **d** are shown in Additional file 1: Figure S2. Here *Cov.* is coverage of screening and *Prev.* the incoming prevalence of CP-CRE

Our primary outcome, the number of CP-CRE days at risk in a year, was lowest for the (A) Direct PCR screening algorithm under all scenarios (Table 3, Fig. 3a). Under the high prevalence scenario, with 63% screening coverage, there were 5080 (standard deviation, SD of 36), 6664 (42) and 5194 (31) days at risk under the screening algorithms (A) Direct PCR, (B) Culture + PCR and (C) PHE respectively (Table 3, Fig. 3a). These high levels were reduced considerably by increasing the screening coverage to 100% (Table 3, Fig. 3a).

**Table 3:** Results table

Screening algorithm	Scenario			Outcomes				
	Speciality	Screening coverage	CP-CRE prevalence	Number of “days at risk”	Total isolation bed days	Total isolation bed days of patients without CP-CRE	Cost per risk day averted (£)	Average incremental cost per additional averted risk day (£)
(A) Direct PCR	ICU	100%	1.6%	90 (4.39)	1500 (19.25)	368 (94.96)	198.45	743.56
		63%	1.6%	508 (14.83)	991 (17.98)	244 (72.30)	192.18	712.38
		63%	20%	5080 (36.20)	**7649** (50.40)	173 (58.47)	58.18	97.10
		100%	20%	918 (14.39)	**11,834** (49.99)	263 (85.91)	58.69	99.57
(B) Culture +PCR		100%	1.6%	335 (9.31)	910 (17.59)	3 (5.55)	63.05	–
		63%	1.6%	642 (14.06)	600 (15.84)	4 (10.01)	61.38	–
		63%	20%	6664 (42.32)	**5955** (38.82)	17 (20.84)	48.09	–
		100%	20%	3308 (24.68)	**9282** (53.2)	29 (38.32)	48.44	–
(C) PHE		100%	1.6%	221 (3.74)	1024 (19.37)	28 (31.95)	83.18	288.63
		63%	1.6%	655 (14.00)	623 (17.57)	13 (18.32)	78.69	819.56
		63%	20%	5194 (31.49)	**7465** (45.2)	17 (21.47)	48.05	47.90
		100%	20%	2309 (11.15)	**10,287** (51.71)	41 (38.09)	49.68	61.23

The outcomes are given as the mean (standard deviation) from 100 simulations. The cost per risk day averted is the mean total cost divided by the mean number of “days at risk”. Total isolation bed days shown in bold text are greater than the existing total number of isolation bed days available to the ICU at ICHNT

Our secondary outcome, the total number of isolation bed days, was highest for the (A) Direct PCR screening algorithm (991–11,834 across the scenarios, Table 3), many of which were inappropriate (due to isolation of patients without CP-CRE, i.e. many PCR false positives) (Table 3, Fig. 3c). In the high prevalence, high screening scenario, between 254% and 324% of the 3650 annual ICU isolation bed days at ICHNT would be required, simply for CP-CRE. In the ICU scenario with low prevalence and low screening coverage (63%), changing to (A) Direct PCR (from (B) Culture + PCR) would increase the percentage of the annual isolation bed days for the ICU required for CP-CRE from 16% to 27%.

Our third outcome, the total costs associated with each algorithm, highlights the higher cost, from the hospital perspective, of (A) Direct PCR screening (Fig. 3d, Additional file 1: Table S3) and the increasing contribution of isolation costs as prevalence increases. Note that for (A) Direct PCR screening costs are greater than isolation costs at low prevalence.

For our fourth outcome, the mean cost per risk day averted for patients with CP-CRE per year, the pattern was reversed (Fig. 3b). The lowest value was for our (B) Culture + PCR algorithm (range across the four scenarios of £48–£63) and highest for (A) Direct PCR (£59–£198) (Table 3), despite a reduced total number of days at risk under the latter (Fig. 3a). The main driver of changes in this outcome was the prevalence, with the two low prevalence scenarios resulting in high costs per CP-CRE carrier risk day averted, i.e. one to three times higher than in the high prevalence (20%) scenario.

The average incremental cost associated with 1 additional averted risk day for algorithms (A) and (C) against the comparison algorithm (B) was between £97 and £744 and £48 and £820 respectively (Table 3).

### Isolation bed day capacity effects

The “days at risk (adjusted for isolation capacity)” value was higher than our primary outcome only for the high prevalence scenarios (Additional file 1: Table S4) at ~ 8900. In particular, for the high prevalence, high screening coverage scenario, taking into account the isolation bed day capacity limits results in a 9.6 times increase in the number of days at risk.

Using this new “days at risk (adjusted for isolation capacity)” value, the new costs per risk day averted were higher for the high prevalence scenarios, with no difference in the relative comparison between the scenarios ((B) < (C) < (A)), although the relative difference between (B) and (C) became greater (Additional file 1: Table S4).

### Other specialities

The results for the other specialities highlight the importance of variance in coverage and prevalence (Additional file 1: Figure S2 and Tables S5–S8). The vascular speciality was associated with substantially lower numbers of risk days, due to the low CP-CRE prevalence (0.4%), whilst renal and haematology specialities showed substantially higher numbers of days at risk than the ICU (base case) due to higher prevalence and longer average lengths of stay respectively. The low prevalence of CP-CRE in the vascular speciality also had increased costs per averted CP-CRE risk day compared to the ICU, whilst the other specialities had similar values to those from the ICU. The patterns of relative algorithm performance, described for the ICU above, were robust across these specialities.

### Other isolation strategies

Isolation of all patients confirmed to carry a CRE would result in many inappropriate isolation days (i.e. isolation of someone without CP-CRE) under the (C) PHE algorithm due to the high false positive rate resulting from the multiple tests (Additional file 1: Figures S3–S5), about five times higher than under the other algorithms. With a no isolation strategy, it can be seen that there would be approximately 1200 and 15,000 CP-CRE “at risk” patient days under existing or high CP-CRE prevalence respectively (Additional file 1: Figure S5).

### Sensitivity analysis

Reducing the sensitivity of our PCR to 60% to account for a change in genetic marker results in a substantially higher primary outcome (from ~ 500 to ~ 750 days at risk) and increased final outcome (~ £200 vs. ~ £250) for the (A) Direct PCR algorithm (Additional file 1: Figure S6).

### Fit to data

**Fig. 4:**
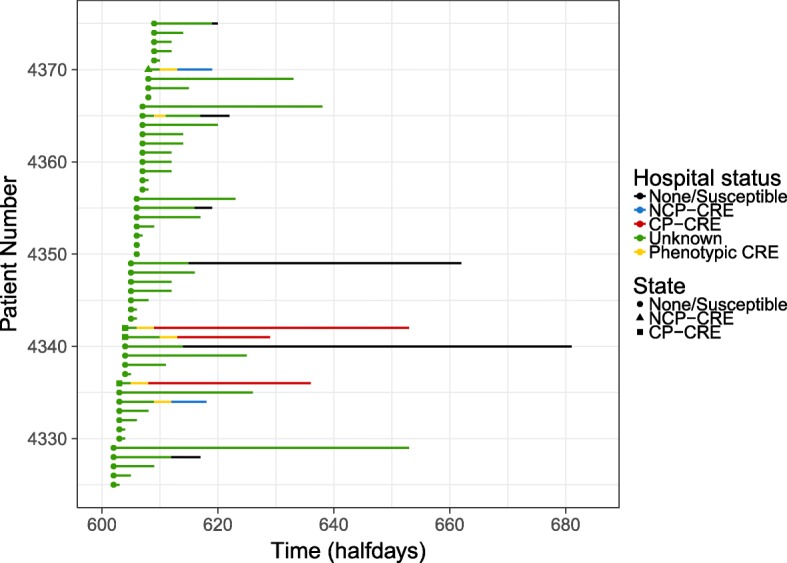
Example model output showing how hospital status changes over a year for patients 4225 to 4375 in one run for the ICU speciality with the (C) PHE screening algorithm. Here each *line* represents a single patient, with *colours* showing how their hospital status changed over time. See Additional file 1: Figure S11 for all patients

Our model accurately recreated the LoS distributions from the data, providing assurance that the model was functioning as anticipated (Additional file 1: Figures S7–S10). For the ICU, there were between 4000 and 5200 patients in the speciality over the year. An example of how the model captures the changing status of a patient over time is shown in Fig. 4, matching the algorithm given in Fig. 1.

## Discussion

We estimated the number of CP-CRE days at risk to be lowest when using our (A) Direct PCR algorithm, owing to the rapidity of result confirmation by PCR. This applied to all scenarios, but was most pronounced in the scenarios with 100% screening coverage levels. However, the false positive rate of the (A) Direct PCR algorithm (a direct false positive rate of 1% vs. an effective false positive of 0.01% for (B) Culture + PCR) resulted in a higher number of inappropriate isolation days and hence substantially higher costs per CP-CRE carrier risk day averted and total costs for the (A) Direct PCR algorithm. This meant that, in terms of cost per CP-CRE carrier risk day averted, the (B) Culture + PCR algorithm performed best.

The differences between algorithms were reduced under scenarios with high prevalence, when the cost per CP-CRE carrier risk day averted was similar for all three algorithms, independently of screening prevalence. This highlights the importance of local epidemiology on determining the impact of screening algorithms. At ICHNT, the screening coverage has risen considerably since the study was performed (now at 96% for the ICU) due to quality improvement work in the trust. This suggests that for the ICHNT ICU setting, where CP-CRE prevalence is low but near 100% coverage (similar to our baseline scenario), the (A) Direct PCR algorithm would give the smallest number of days at risk. However, the (B) Culture + PCR algorithm would be substantially better in terms of cost per CP-CRE carrier risk day averted. The (C) PHE algorithm, which is basically three repeats of the (B) Culture + PCR algorithm with an additional high-performance PCR, performs slightly better in terms of risk days averted than (B) Culture + PCR; however, the cumulative costs of these repeats result in a higher cost per CP-CRE carrier risk day averted. This is reflected in substantial average incremental costs, suggesting that the ICHNT should continue to use (B) Culture + PCR.

The same pattern of algorithm performance is seen in the three other ICHNT specialities considered, which have CP-CRE prevalence on admission ranging from 0.4 to 1.9%, but different bed numbers and lengths of stay. Data on CP-CRE admission prevalence across England (0.1% from another London hospital in 2015 [21]) and Europe (1.1% in a Spanish hospital in 2006–2010 [31]) are scarce, but the level is likely to lie within the range considered here (< 8%) [29]. Thus, the cost per CP-CRE carrier risk day averted is likely to be lowest for (B) Culture + PCR in other English settings. Only as prevalence increases will the false positive rate of Direct PCR algorithms be counterbalanced, and the use of multiple screens or a direct-from-swab PCR have decreasing cost per CP-CRE carrier risk day averted. This is similar to cost-effectiveness results from the USA, where culture-based algorithms were found to be cost-effective, whilst PCR gave much higher costs [32].

The impact of these screening algorithms on the demand for isolation bed days rapidly increases with both screening coverage and CP-CRE prevalence. Isolation beds (side rooms) are in demand for other uses than CP-CRE, and thus even before the > 100% demand under all the high CP-CRE (20%) prevalence scenarios is seen, such requirements may not be available. However, increasing screening coverage is very important in reducing the number of days at risk (and hence the potential for onward transmission). In our case, increasing screening coverage from 63 to 100% reduced the number of days at risk by more than 50%. This, however, was only true when we ignored the limits of the existing isolation bed day capacity. When we included this, the number of days at risk was similar, as the limit on the days at risk was not screening coverage but isolation bed availability. Thus, pairing increased screening coverage with concurrent isolation bed day availability should be a focus of hospitals [25], especially as CP-CRE prevalence on admission rises. Alternatives such as nurse cohorting or increased contact precautions for certain patients, instead of speciality isolation beds, could also be employed. Laboratory capacity should also be considered under increased prevalence demands, as well as screening outside of only high-risk areas, neither of which we included here.

The rapidity of direct PCR tests or equivalent “point-of-care” rapid diagnostic tests makes them an attractive option for hospitals. However, their cost and the impact on isolation bed day capacity cannot be ignored. As CP-CRE prevalence increases, this cost would be reduced due to the lower proportion of false positives and the rapidity of detection. Our costings include the extra cost generated from a patient isolated following a false positive test. We considered only existing technology; however, future diagnostic tests for CP-CRE including “lab on a chip” mechanisms would be rapid and highly specific. As shown here, if these tests can improve on the false positive detection rate of PCR, potentially by combining phenotypic and genotypic output, then at a low cost, these could be greatly improved algorithms, reducing dramatically the number of days at risk.

The clinical impact of our results is to provide evidence for hospitals to decide between screening strategies for detection and isolation of CP-CRE carriers to prevent ongoing transmission. One aspect of the screening algorithms modelled in this paper that is not captured in the outcomes that we evaluated is that the Direct PCR algorithm would result in more rapid identification of the specific carbapenemase involved: this has value for the rapid identification of potential clusters and understanding short-term local epidemiological trends. In addition, these screening results, in the identification of CP-CRE carriage, can aid in the design of antimicrobial treatment if subsequent infection occurs (or is present already at admission). Whilst the greatest clinical impact would be achieved by the most rapid test, clinical settings operate with strict budgets, and thus a comparison such as those presented here must be made for on-the-ground decision making.

This study’s main strength is its direct linkage to a “real” hospital setting. This leading London teaching hospital group, with 15,000 admissions a month and 1300 beds, provided screening coverage data, CP-CRE prevalence by speciality and LoS data, making the modelling outputs based on “real” data, rather than hypothesised parameters. This makes the applicability of the model better and supports the reliability of the results.

The main weakness of this study was that transmission was not explicitly included in the model due to a lack of reliable estimates for CP-CRE transmission rates. Therefore, the effects of isolation on CPE prevalence could not be explored, and we do not capture the indirect impact of these screening algorithms. However, the number of CP-CRE days at risk is a proxy for heightened levels of transmission, and our comparison of screening tests would be similar with the addition of indirect transmission effects, although the likely impact may change, potentially non-linearly, with increasing resistance prevalence. There is likely to be considerable uncertainty in the transmission rate from individual patients (those isolated and not) and between settings, making the addition of this complexity unlikely to clarify or improve on our results. Such “colonisation” burden proxies of transmission risk have been proposed before [33], with analysis showing that they link directly to acquisition rates of other resistant pathogens [34, 35]. Other weaknesses, in terms of modelling assumptions, come from our assumption that those with CP-CRE and NCP-CRE could be grouped as having the same LoS distribution, i.e. that those with CRE have the same LoS. Apart from CRE status, other bacterial and all host heterogeneities were missing, such as risk factors for carriage. We also did not undertake a cost-effectiveness analysis, instead looking at the overall effect of different variables on the impact of screening algorithms. In addition, although we explored implementation through exploring the impact of limited numbers of isolation bed days, we did not include further financial or technical constraints.

There are also limitations to our cost calculations, in particular, the specific nature of our parameterisation, which makes the costing results specific to our setting and does not include the variation that may be seen. This limits the generalisability to broad conclusions about the comparative nature of the algorithms. We also decided to use “cost per CP-CRE carrier risk day averted” as the main cost comparison method. As we did not include transmission, we could not include the cost of CP-CRE outbreaks, and instead only the proxy of “risk days”. In terms of clinical impact, time to detection of CP-CRE may override this cumulative “risk day” calculation (if we believe that transmission from a patient could saturate), but we believe that this is unlikely due to rapid patient movement and therefore the same ongoing transmission risk from every “risk day”. Thus rapidity of test result may not be optimal when making difficult value-based decisions for infection control.

The key next step for this work depends on an improved understanding of the transmission routes and pathways that lead to patients being carriers of CP-CRE, as well as the length of time patients are carriers. Once this is available, adding in transmission, and the effect of isolation on transmission, would allow for estimation of the impact on the additional indirect effects of screening. Similarly, with information on the quantitative impact of different risk factors on CP-CRE carriage (e.g. by what relative increase are those who travel abroad more likely to carry CP-CRE) and the prevalence of these risk factors, more heterogeneity in the patient host population could be included.

## Conclusions

In conclusion, in English settings, where CP-CPE prevalence is still low, we would recommend continuation of Culture + PCR-based algorithms, despite the reduction in days at risk if a Direct PCR algorithm were introduced. The latter suffers from a higher false positivity rate, which results in an unacceptably high cost per risk day averted whilst CP-CRE prevalence remains low.

## Additional file

Additional file 1: Additional Methods (length of stay calculations, cost calculations and details of algorithms) and Additional Results (total costs, isolation bed day capacity effects, outcomes for all four specialities, results for all isolation strategies, sensitivity analyses, length of stay match to data and individual patient trajectories). (DOCX 2129 kb)

## Abbreviations

CDC: Centers for Disease Control and Prevention
CP-CRE: Carbapenemase-producing carbapenem-resistant Enterobacteriaceae
CRE: Carbapenem-resistant Enterobacteriaceae
ECDC: European Centre for Disease Prevention and Control
ESCMID: European Society of Clinical Microbiology and Infectious Diseases
ICHNT: Imperial College Healthcare NHS Trust
ICU: Intensive care unit
LoS: Length of stay
NCP-CRE: Non-carbapenemase-producing carbapenem-resistant Enterobacteriaceae
PCR: Polymerase chain reaction
PHE: Public Health England
