# Supplementary material for: Fast and expensive (PCR) or cheap and slow (culture)? A mathematical modelling study to explore screening for carbapenem resistance in UK hospitals
Source: BMC Med. 2018 Aug 16;16:141. doi: 10.1186/s12916-018-1117-4 (PMC6094916; doi:10.1186/s12916-018-1117-4)
Supplement: Supplementary file 1 — Additional Methods (length of stay calculations, cost calculations and details of algorithms) and Additional Results (total costs, isolation bed day capacity effects, outcomes for all four specialities, results for all isolation strategies, sensitivity analyses, length of stay match to data and individual patient trajectories). (DOCX 2129 kb) [file 12916_2018_1117_MOESM1_ESM.docx]

**Supplementary for:** Fast and expensive (PCR) or cheap and slow (culture)? A mathematical modelling study to explore screening for carbapenem resistance in UK hospitals

**Contents**

***Additional methods***

1. Length of stay data calculations
2. Cost calculations
3. Details of algorithms

***Additional results***

1. Total costs
2. Isolation bed day capacity effects
3. Outcomes for all four specialities (ICU, Renal, Haematology, Vascular)
4. Results for all isolation strategies
5. Outcomes of sensitivity analysis
6. Length of stay match to data
7. Individual patient trajectories

***Additional methods***

1. **Length of stay data calculations**

*Length of stay parameter estimation for CRE positive and CRE negative patients in ICU, Renal, Vascular and Haematology wards*

We analysed the episodes of the length of stay of a population of patients that were admitted to Imperial College Healthcare NHS Trust and had a CRE test. The time period from June 2015 to March 2016 was covered, where CRE universal screening specimens were considered. Due to frequent patients’ movement across different specialties, the length of stay of each patient was divided by ward and only ICU, Renal, Vascular and Haematology wards were selected for analysis. The data of the defined population test results was analysed. We searched for presence of any CRE.

Due to the nature of the in-house tests, which involves plating the organisms onto a selective medium specific for detecting resistance to carbapenem, the test results were divided according to five types of antibiotics used to determine resistance, such as Meropenem, Ertapenem, IMP, Tazobactam and Temocillin.

Test results data showed presence of the following organisms:

- Acinetobacter
- Aeromonas and Steno-
- Enterobacteriaceae

We analysed the sensitivity of the above mentioned bacterial families to one or more of the antibiotics used for testing (Meropenem, Ertapenem, IMP, Tazobactam and Temocillin). Patients with resistant and intermediate resistant results were grouped together and considered to be CRE positive. All CRE culture positive results were confirmed using in-house PCR test and in case of uncertainty regarding the resistance mechanism they were sent to PHE reference laboratory for confirmation. CRE negative group consisted of all other results, which included patients with no CRE present.

We calculated the average length of stay for both groups and found out that CRE positive patients’ length of stay is significantly higher than CRE negative patents’ length of stay (Table S1).

|  | Number of spells* with CRE(+ve) | Number of spells with CRE(-ve) | Average LoS CRE+, days | Average LoS CRE-, days | Difference,% |
| --- | --- | --- | --- | --- | --- |
|  |  |  |  |  | (CRE+ – CRE-)/(CRE+) |
| ICU | 85 | 2337 | 15.9 | 7.9 | 50% |
| Renal | 166 | 1668 | 15.5 | 7.8 | 50% |
| Vascular | 62 | 1750 | 12.4 | 6.2 | 50% |
| Haematology | 39 | 973 | 19.6 | 12.9 | 34% |

Table S1: figures for length of stay calculations

1. **Cost calculations**

We used cost calculations from ICHNT (personal communication) alongside those for ICHNT published by Otter *et al.* (Otter, 2016).

| Screening test | Components | Staff cost | Cost of screening | Total |
| --- | --- | --- | --- | --- |
| Culture | Culture test 1 | £1.22 | £1 | £2.22 |
|  | Culture sensitivity test (CRE prevalence x 2) | / | £8 | £8.00 |
| ICHNT PCR | In-house PCR test | £8.74 | £20 | £28.74 |
| PHE PCR |  | / | / | £0 |

Table S2: Details of costs included in the model.

1. **Details of all screening algorithms**

**

Figure S1: Details of all stages for patients across the three screening algorithms. In the third (C) PHE algorithm, a * means a transfer to PHE for testing with their PCR.

***Additional results***

1. **Total costs**

The total costs (£) for all the scenarios investigated is given below (Table S3). These are calculated as the sum of the screening costs and isolation costs for each scenario.

| **Isolation strategy** | **Screening scenario** | **Scenarios** | | | | **Speciality** | | |
| --- | --- | --- | --- | --- | --- | --- | --- | --- |
|  |  | **ICU**  **(high coverage)** | **ICU (ICHNT)** | **ICU**  **(high prev.)** | **ICU**  **(high cov. and prev.)** | **Renal** | **Haematology** | **Vascular** |
| No-one | Direct PCR | 146411 [140337,154679] | 92747 [87686,98118] | 75947 [71505,80673] | 119676 [112603,127174] | 60233 [57221,63515] | 36276 [34316,38397] | 49651 [46904,53399] |
|  | Culture+PCR | 17630 [16069,19394] | 11168 [10052,12382] | 25227 [23019,27825] | 39533 [36473,43138] | 9809 [8341,11173] | 4693 [3940,5571] | 5611 [4894,6428] |
|  | PHE | 37813 [34448,41440] | 21005 [18634,22681] | 34916 [31815,37607] | 58307 [52924,62508] | 17462 [15565,19345] | 10808 [9443,12308] | 9215 [7932,10474] |
| Those with CP-CRE* | Direct PCR | 224764 [200489,254214] | 143411 [121878,168192] | 434962 [388727,488636] | 679098 [624459,741794] | 80191 [69873,95389] | 47731 [39652,58009] | 55048 [48579,65148] |
|  | Culture+PCR | 57199 [40622,75363] | 36604 [24498,56464] | 285555 [251011,331427] | 448260 [393768,511639] | 21575 [12972,32354] | 10388 [5075,24556] | 7384 [4894,14491] |
|  | PHE | 82863 [61930,104540] | 48021 [32856,67880] | 357896 [314378,408825] | 509061 [446577,561945] | 30505 [22204,46282] | 17185 [10506,27013] | 10781 [8055,15162] |
| Those with CRE | Direct PCR | 224764 [200489,254214] | 143411 [121878,168192] | 434962 [388727,488636] | 679098 [624459,741794] | 80191 [69873,95389] | 47731 [39652,58009] | 55048 [48579,65148] |
|  | Culture+PCR | 86847 [66709,107900] | 55666 [40758,76291] | 367773 [321906,416975] | 573420 [516032,641429] | 51777 [34109,68536] | 17642 [9133,33856] | 13545 [8908,21101] |
|  | PHE | 224600 [184348,270090] | 121012 [91715,148384] | 474246 [425432,525000] | 718550 [643435,772171] | 81122 [67225,102456] | 46564 [34403,65105] | 24071 [16245,33925] |

Table S3: Total costs (£) (mean and range) for each scenario and speciality. * These results are shown in Table 3 & Figure 3 for the ICU scenarios.

1. **Isolation bed day capacity effects**

Including the number of “detected but not isolated” patient bed days in the number of “days at risk (adjusted for isolation capacity)” calculation shows that only at high CP-CRE prevalence would the number of isolation bed days be insufficient. However, this does not take into account isolation bed day demand for other purposes.

Cost per risk day averted (adjusted for isolation capacity) was calculated by subtracting from the original total costs the costs due to the individual isolation days that were not available. We made no assumptions about which patients would ever be isolated and so this cost included the one-off isolation costs (i.e. assumed that every detected patient would at some point be isolated).

| **Screening algorithm** | **Scenario** | | |  | |  |
| --- | --- | --- | --- | --- | --- | --- |
|  | **Speciality** | **Screening coverage** | **CP-CRE prevalence** | **Number of**  **“days at risk”** | **Number of**  **“days at risk (adjusted for isolation capacity)”** | **Cost per risk day averted (£)** |
| 1. Direct PCR | ICU | 100% | 1.6% | 90 (4.39) | 90 (19.01) | 198.45 |
|  |  | 63% | 1.6% | 508 (14.83) | 508 (22.33) | 192.18 |
|  |  | 63% | 20% | 5080 (36.2) | 8906 (62.24) | 97.85 |
|  |  | 100% | 20% | 918 (14.39) | 8840 (51.3) | 141.95 |
| 1. Culture +PCR |  | 100% | 1.6% | 335 (9.31) | 335 (19.83) | 63.05 |
|  |  | 63% | 1.6% | 642 (14.06) | 642 (21.14) | 61.38 |
|  |  | 63% | 20% | 6664 (42.32) | 8952 (57.38) | 65.50 |
|  |  | 100% | 20% | 3308 (24.68) | 8911 (58.48) | 91.60 |
| 1. PHE |  | 100% | 1.6% | 221 (3.74) | 221 (19.29) | 83.18 |
|  |  | 63% | 1.6% | 655 (14) | 655 (22.51) | 78.69 |
|  |  | 63% | 20% | 5194 (31.49) | 8992 (55.18) | 76.90 |
|  |  | 100% | 20% | 2309 (11.15) | 8905 (53.43) | 102.73 |

Table S4: Additional results table. The outcomes are given as the mean (standard deviation) from 100 simulations.

1. **Outcomes for all four specialities**

Additional results by the four main specialities where universal screening was in place at ICHNT (ICU, Renal, Haematology and Vascular) are given below. Note here that ICU is the ICU (ICHNT) scenario in the main scenario analysis: 64% coverage of screening and 1.6% prevalence. The same results figure and table as in the main paper are given first for the four main specialities (Figure S2 and Table S5).

The results broken down by the four specialities for the three isolation scenarios are given below for the baseline scenario of isolating anyone with a CP-CRE (Table S5), anyone with a CRE (Table S6) and isolating no-one (Table S7).

Figure S2: Results figure for the four specialities showing our two main outcomes: A), number of days at risk and B) cost per CP-CRE carrier risk day averted. Additional outcomes of inappropriate isolation days C) and Total costs (£) D) are also shown. Error bars in A are standard error. No error bars are shown in B as this is the ratio of means. Details of the errors for values in C&D are shown in Table S5. Here “Haem.” is Haematology.

| **Screening algorithm** | **Scenario** | | | | | **Primary outcome** | | | **Secondary outcome** |
| --- | --- | --- | --- | --- | --- | --- | --- | --- | --- |
|  | **Speciality** | **Screening coverage** | **Length of stay**  **(S / CRE)** | **Number of beds** | **CP-CRE prevalence** | **Number of “days at risk”** | **Total isolation bed days** | **Total isolation bed days of patients without CRE** | **Cost per risk day averted (£)** |
| 1. Direct PCR | ICU | 63.0% | 7.9 / 15.9 | 112 | 1.6% | 508 (14.83) | 991 (17.98) | 244 (7.23) | 192.18 |
|  | Renal | 67.0% | 7.8 / 15.5 | 71 | 1.9% | 333 (12.19) | 665 (14.39) | 141 (4.25) | 156.13 |
|  | Haematology | 68.0% | 6.2 / 12.4 | 66 | 1.3% | 159 (10.81) | 397 (12.87) | 149 (6.16) | 191.83 |
|  | Vascular | 48.0% | 9.6 / 19.6 | 65 | 0.4% | 93 (6.26) | 173 (6.64) | 99 (3.84) | 757.89 |
| 1. Culture +PCR | ICU | 63.0% | 7.9 / 15.9 | 112 | 1.6% | 642 (14.06) | 600 (15.84) | 3 (0.95) | 61.38 |
|  | Renal | 67.0% | 7.8 / 15.5 | 71 | 1.9% | 448 (12.62) | 426 (13.85) | 1 (0.37) | 52.2 |
|  | Haematology | 68.0% | 6.2 / 12.4 | 66 | 1.3% | 198 (10.68) | 224 (12.82) | 1 (0.31) | 47.63 |
|  | Vascular | 48.0% | 9.6 / 19.6 | 65 | 0.4% | 113 (6.61) | 63 (5.19) | 1 (0.39) | 126.88 |
| 1. PHE | ICU | 63.0% | 7.9 / 15.9 | 112 | 1.6% | 655 (14) | 623 (17.57) | 11 (1.64) | 78.69 |
|  | Renal | 67.0% | 7.8 / 15.5 | 71 | 1.9% | 425 (9.15) | 425 (11.67) | 5 (0.99) | 72.3 |
|  | Haematology | 68.0% | 6.2 / 12.4 | 66 | 1.3% | 193 (9.45) | 225 (12.65) | 10 (1.47) | 78.75 |
|  | Vascular | 48.0% | 9.6 / 19.6 | 65 | 0.4% | 97 (5.65) | 59 (3.94) | 4 (0.8) | 203.36 |

Table S5: Results table. Values in brackets are the standard deviation from 100 simulations. The secondary outcome is the mean total cost divided by the mean total number of days CP-CRE carriers are isolated.

| **Speciality** | **Screening scenario** | **Total isolation bed days** | **Total isolation bed days of patients with CP-CRE** | **Total isolation bed days of patients without CRE** | **Total days those patients with CP-CRE are not isolated** | **Percentage of total isolation bed days for patients with CP-CRE** |
| --- | --- | --- | --- | --- | --- | --- |
| ICU | Direct PCR | 991 [584,1420] | 746 [440,1186] | 244 [108,502] | 508 [212,916] | 75 [57,91] |
|  | Culture+PCR | 600 [275,1202] | 596 [275,1202] | 3 [0,56] | 642 [334,1132] | 99 [92,100] |
|  | PHE | 623 [264,1238] | 610 [262,1232] | 11 [0,67] | 655 [398,1058] | 98 [87,100] |
| Renal | Direct PCR | 665 [404,1140] | 512 [272,1002] | 141 [41,255] | 333 [137,828] | 77 [54,95] |
|  | Culture+PCR | 426 [224,1136] | 419 [224,1136] | 1 [0,18] | 448 [206,947] | 98 [89,100] |
|  | PHE | 425 [143,796] | 410 [126,783] | 5 [0,48] | 425 [244,675] | 96 [80,100] |
| Haematology | Direct PCR | 397 [96,776] | 248 [37,564] | 149 [32,346] | 159 [22,512] | 61 [33,91] |
|  | Culture+PCR | 224 [16,592] | 223 [16,592] | 1 [0,22] | 198 [38,546] | 100 [85,100] |
|  | PHE | 225 [52,662] | 214 [50,657] | 10 [0,86] | 193 [42,459] | 95 [67,100] |
| Vascular | Direct PCR | 173 [46,358] | 73 [4,275] | 99 [28,220] | 93 [4,361] | 39 [5,83] |
|  | Culture+PCR | 63 [0,216] | 58 [0,216] | 1 [0,24] | 113 [7,326] | 93 [0,100] |
|  | PHE | 59 [0,175] | 54 [0,172] | 4 [0,44] | 97 [21,350] | 90 [0,100] |

Table S6: Secondary outcomes (total number of isolation bed days) required for isolation scenario of isolating anyone with a CP-CRE.

| **Speciality** | **Screening scenario** | **Total isolation bed days** | **Total isolation bed days of patients with CP-CRE** | **Total isolation bed days of patients without CRE** | **Total days those patients with CP-CRE are not isolated** | **Percentage of total isolation bed days for patients with CP-CRE** |
| --- | --- | --- | --- | --- | --- | --- |
| ICU | Direct PCR | 991 [584,1420] | 746 [440,1186] | 244 [108,502] | 508 [212,916] | 75 [57,91] |
|  | Culture+PCR | 925 [564,1496] | 609 [282,1202] | 253 [101,484] | 629 [334,1128] | 66 [42,87] |
|  | PHE | 1943 [1246,2736] | 621 [266,1232] | 1247 [682,1702] | 645 [398,1046] | 32 [16,52] |
| Renal | Direct PCR | 665 [404,1140] | 512 [272,1002] | 141 [41,255] | 333 [137,828] | 77 [54,95] |
|  | Culture+PCR | 1467 [1090,2096] | 431 [224,1138] | 148 [48,274] | 435 [206,942] | 29 [16,58] |
|  | PHE | 2067 [1688,2901] | 418 [126,783] | 703 [482,978] | 417 [230,662] | 20 [6,34] |
| Haematology | Direct PCR | 397 [96,776] | 248 [37,564] | 149 [32,346] | 159 [22,512] | 61 [33,91] |
|  | Culture+PCR | 470 [174,1044] | 227 [16,619] | 170 [57,410] | 194 [38,455] | 46 [8,84] |
|  | PHE | 1199 [762,1796] | 222 [60,657] | 899 [589,1318] | 186 [42,459] | 18 [5,39] |
| Vascular | Direct PCR | 173 [46,358] | 73 [4,275] | 99 [28,220] | 93 [4,361] | 39 [5,83] |
|  | Culture+PCR | 233 [83,548] | 60 [0,258] | 98 [28,242] | 112 [7,326] | 24 [0,66] |
|  | PHE | 444 [282,734] | 55 [0,172] | 317 [206,525] | 97 [21,350] | 12 [0,37] |

Table S7: Secondary outcomes (total number of isolation bed days) required for isolation scenario of isolating anyone with a CRE.

| **Speciality** | **Screening scenario** | **Total isolation bed days** | **Total isolation bed days of patients with CP-CRE** | **Total isolation bed days of patients without CRE** | **Total days those patients with CP-CRE are not isolated** | **Percentage of total isolation bed days for patients with CP-CRE** |
| --- | --- | --- | --- | --- | --- | --- |
| ICU | Direct PCR | 0 [0,0] | 0 [0,0] | 0 [0,0] | 1254 [687,1824] | 0 [0,0] |
|  | Culture+PCR | 0 [0,0] | 0 [0,0] | 0 [0,0] | 1238 [870,1706] | 0 [0,0] |
|  | PHE | 0 [0,0] | 0 [0,0] | 0 [0,0] | 1266 [862,1836] | 0 [0,0] |
| Renal | Direct PCR | 0 [0,0] | 0 [0,0] | 0 [0,0] | 845 [482,1633] | 0 [0,0] |
|  | Culture+PCR | 0 [0,0] | 0 [0,0] | 0 [0,0] | 866 [504,1522] | 0 [0,0] |
|  | PHE | 0 [0,0] | 0 [0,0] | 0 [0,0] | 835 [455,1292] | 0 [0,0] |
| Haematology | Direct PCR | 0 [0,0] | 0 [0,0] | 0 [0,0] | 407 [138,916] | 0 [0,0] |
|  | Culture+PCR | 0 [0,0] | 0 [0,0] | 0 [0,0] | 421 [103,961] | 0 [0,0] |
|  | PHE | 0 [0,0] | 0 [0,0] | 0 [0,0] | 408 [155,918] | 0 [0,0] |
| Vascular | Direct PCR | 0 [0,0] | 0 [0,0] | 0 [0,0] | 166 [32,373] | 0 [0,0] |
|  | Culture+PCR | 0 [0,0] | 0 [0,0] | 0 [0,0] | 171 [7,420] | 0 [0,0] |
|  | PHE | 0 [0,0] | 0 [0,0] | 0 [0,0] | 151 [38,368] | 0 [0,0] |

Table S8: Secondary outcomes (total number of isolation bed days) required for isolation scenario of isolating no-one.

1. **Results for all isolation strategies**

The below figures give the additional plots of the secondary outcomes for the baseline isolation strategy of isolating anyone with CP-CRE (Figure S3), as well as two others: isolating anyone confirmed to carry CRE (Figure S4) or isolation of no-one (Figure S5).

In the baseline isolation strategy (isolation of those with CP-CRE), there is a high number of inappropriate isolation of those without CRE under the (A) Direct PCR algorithm (Figure S3).

Under the isolation of all with CRE, more are inappropriately isolated with (C) “PHE” due to the false positive CRE detection (Figure S4). This is due to the compound effect of multiple culture screens across the population. We do not track whether a patient has already had a negative screen, and instead test everyone in each round. These patients are also the ones that will stay in for longer: if screened multiple times and found, then the patient must have been in hospital for a long time and therefore is likely to have a long length of stay.

When no-one is isolated, the only count is those “with CP-CRE no-isolated” (Figure S5). Note that this highlights the baseline number of CP-CRE per bed day in the speciality and that it is a sum of isolation days “with CP-CRE” and “with CP-CRE not-isolated” under the other isolation scenarios.

Figure S3: Isolation day counts per bed in the speciality for an isolation strategy of isolating anyone confirmed to be carrying CP-CRE. Total number of days that those with CP-CRE are isolated or not isolated (top row). Inappropriate isolation (isolation of a patient without CRE) and total isolation days (bottom row). The error bars are the standard error from 100 model simulations.

Figure S4: Isolation day counts per bed in the speciality for an isolation strategy of isolating anyone confirmed to carry a CRE. Total number of days that those with CP-CRE are isolated or not isolated (top row). Inappropriate isolation (isolation of a patient without CRE) and total isolation days (bottom row). The error bars are the standard error from 100 model simulations.

Figure S5: Isolation day counts per bed in the speciality for an isolation strategy of isolating no-one. Total number of days that those with CP-CRE are isolated or not isolated (top row). Inappropriate isolation (isolation of a patient without CRE) and total isolation days (bottom row). The error bars are the standard error from 100 model simulations.

1. **Outcomes of sensitivity analysis**

Results for the sensitivity analyses are shown in Figure S6.

Figure S6: Results figure for the “ICU” scenario with low screening coverage and prevalence, and “low PCR sensitivity” sensitivity analysis showing our two main outcomes: A), number of days at risk and B) cost per CP-CRE carrier risk day averted. Additional outcomes of inappropriate isolation days C) and Total costs (£) D) are also shown. Error bars in A are standard error. No error bars are shown in B as this is the ratio of means.

1. **Length of stay match to data**

The model output of length of stays for patients is shown in grey bars, with the data from ICHNT shown in red lines in Figure S7 - Figure S10 below. From this it can be seen that our model accurately captured the length of stay distributions for each speciality.

Figure S7: Length of stay from model (grey bar) and data (red line) for ICU for patients without CRE (left) and with CRE (right).


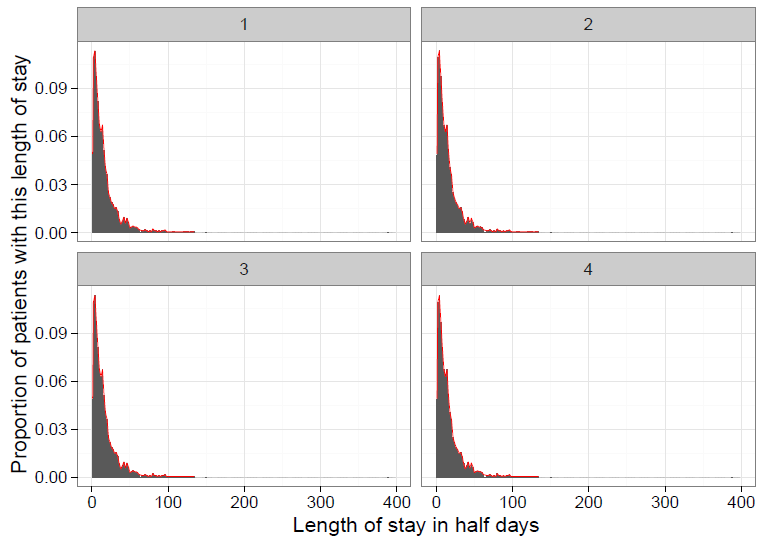

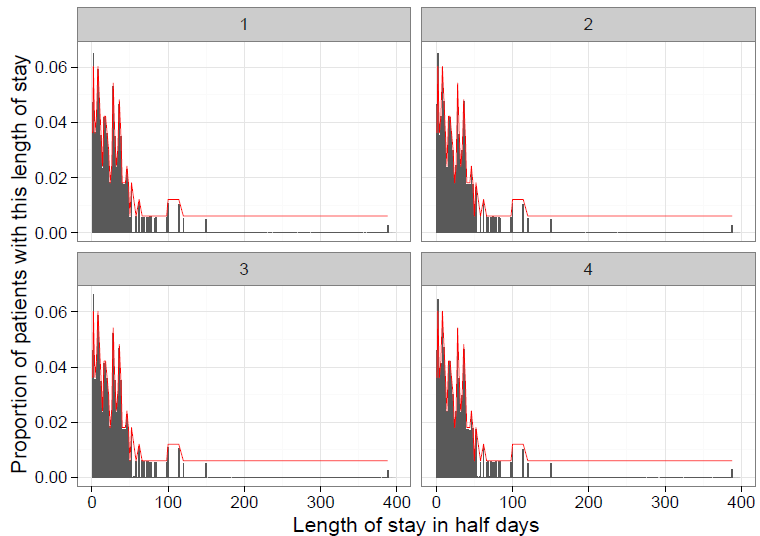


Figure S8: Length of stay from model (grey bar) and data (red line) for renal for patients without CRE (left) and with CRE (right).


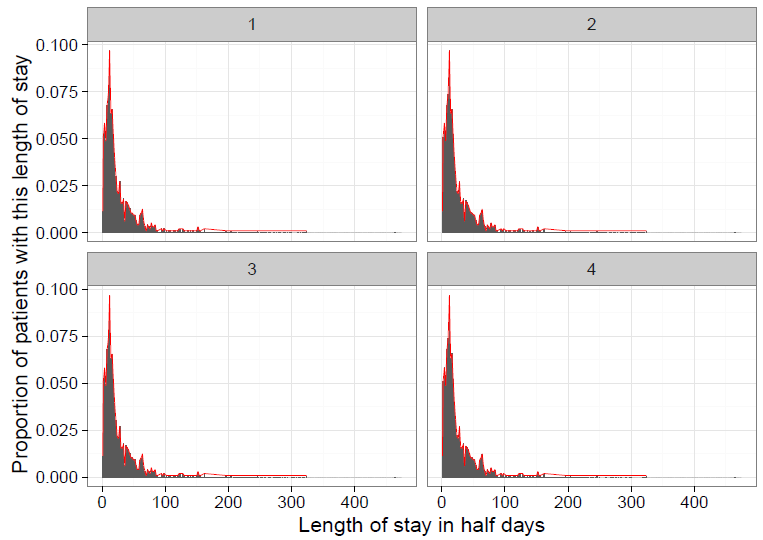

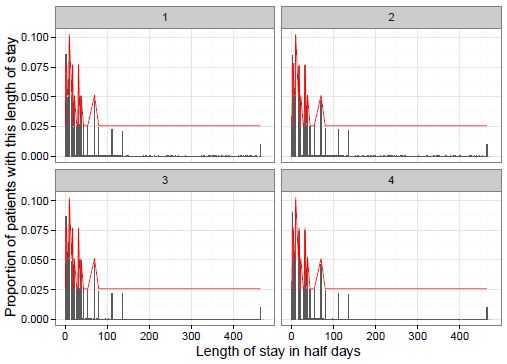


Figure S9: Length of stay from model (grey bar) and data (red line) for haematology for patients without CRE (left) and with CRE (right).


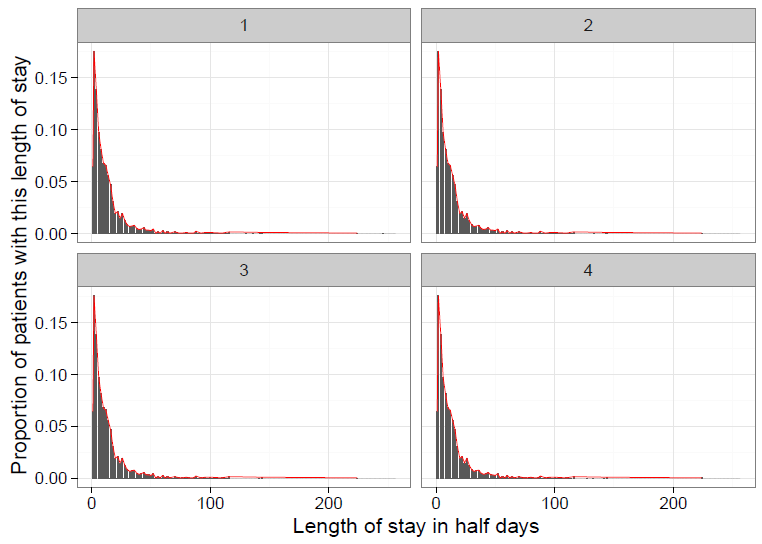

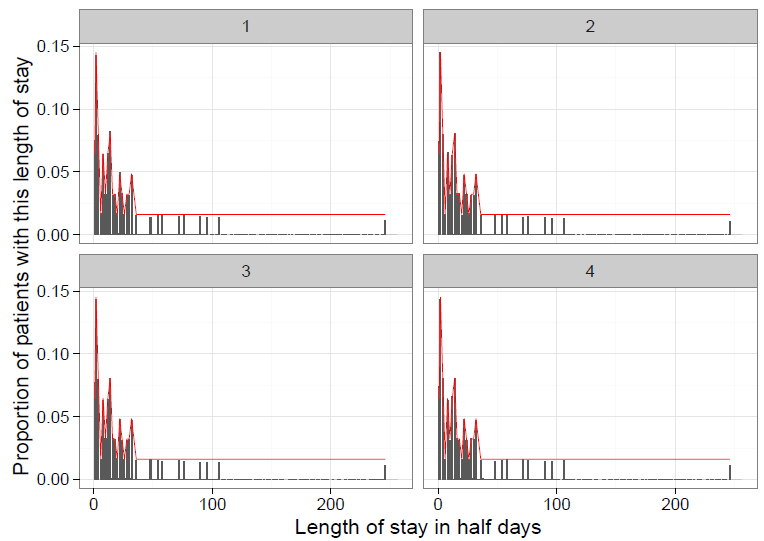


Figure S10: Length of stay from model (grey bar) and data (red line) for vascular for patients without CRE (left) and with CRE (right).

1. **Individual patient trajectories**

Figure 4 in the main paper shows all patient trajectories for the ICU in a year. Figure S11 (below) shows a zoomed in section of this graph, with greater detail on the individual patient bacterial state (what type they carrying) by shape and the corresponding hospital status shown in colour.

Figure S11: Example model output showing how hospital status changes over a year for all individual patients for one run for the ICU speciality with the (C) “PHE” screening algorithm. Here each line represents a single patient, with colours showing how their hospital status changed over time. See main text for a zoomed in example.
